# The impact of coarse aggregate mineral compositions on skid resistance performance of asphalt pavement: A comprehensive study

**DOI:** 10.1371/journal.pone.0308721

**Published:** 2024-12-26

**Authors:** Chen Zhang, Lihao Zeng, Huimin Wang, Xin Qu

**Affiliations:** 1 Guangxi Transportation Science and Technology Group Co., Ltd., Nanning, Guangxi, China; 2 Xi’an Aeronautical Institute, Xi’an, Shaanxi, China; 3 Zhejiang Jiaotou Expressway Construction Management Co., Hangzhou, Zhejiang, China; 4 Key Laboratory of Intelligent Construction and Maintenance of CAAC, Xi’an, Shaanxi, China; Beijing University of Technology, CHINA

## Abstract

This study aimed to investigate the influence of different coarse aggregate mineral compositions on the skid resistance performance of asphalt pavement. The imprint method was utilized to assess the contact probability between various graded asphalt surface aggregates and tires. Additionally, macroscopic adhesive friction coefficients between polished surfaces of three types of rock slabs (basalt, limestone, granite) and rubber were determined using a pendulum friction tester. Molecular dynamics simulations were employed to model the main aggregate minerals and rubber, and a “sandwich” type constrained shear model was constructed to evaluate micro-scale adhesive friction coefficients. Results indicated a 40% contact probability between aggregate and tire in a unit area of the road surface, highlighting the importance of studying adhesive friction between minerals and rubber. Macroscopically, basalt exhibited the highest adhesive friction coefficient, followed by limestone and granite. At the molecular level, feldspar showed the highest micro-scale friction coefficient with rubber, while quartz exhibited the lowest. The micro-scale adhesive friction coefficients correlated well with the macroscopic findings (correlation coefficient of 0.81), providing theoretical support for optimizing coarse aggregate selection to enhance skid resistance in road applications.

## 1. Introduction

China has constructed an impressive network of highways spanning a total length of 5.3548 million kilometers, surpassing all other countries. As a part of road structure, the asphalt surface should not only provide good surface characteristics but also pay more attention to the skid resistance of the road [1, 2]. Both road safety and road performance are issues that need to be addressed during the service life of a road.

Insufficient skid resistance of the road or inadequate skid resistance of the road surface due to repeated effects of vehicles often lead to frequent traffic accidents. It is known that the braking effect of a vehicle is influenced by the adhesion between the tire and the road surface [3]. When the road texture deteriorates and becomes polished, the maximum braking force provided by the road to the vehicle is inevitably reduced, resulting in longer braking distance and increased risk of traffic accidents [4–6]. Reference [7] compiled statistical results on the friction coefficient of roads in Denmark and Norway and the accident rate, and found that there was an inverse correlation between the road friction coefficient and the accident rate. Research has also shown that for every 10% improvement in skid resistance, the accident rate decreases by about 13% [8]. In this context, the skid resistance of the road has become a hot research topic in the road transportation industry.

Aggregate is the main component of asphalt mixture, accounting for as much as 90% [9]. Aggregates can affect the performance of asphalt mixtures, thereby affecting the performance and service life of asphalt pavements [10]. At the same time, the quality of aggregates is one of the important factors affecting the skid resistance of the road surface [11]. So the research objectives involved were mainly asphalt pavements rather than cement concrete pavements. However, the impact of aggregate mineral composition and morphological characteristics on the skid resistance of asphalt pavements is not well studied.

Based on the timeline of the service life of asphalt pavements, it is found that in the early stage of service (6–24 months), a layer of asphalt film adheres to the surface of the aggregate, allowing the tire to directly contact the asphalt film adhered to the rough aggregate on the road surface. In other words, the adhesion friction coefficient between asphalt and rubber is part of the friction coefficient between the road surface and the tire. However, after enduring the effects of long-term natural environment and traffic loads, the asphalt film on the surface of the rough aggregate is peeled off, exposing the coarse aggregate directly to the natural environment and contacting the vehicle tire. The object that provides adhesive friction force for the skid resistance of the road surface changes from asphalt-rubber to aggregate-rubber, and the interaction time between aggregate and rubber is much longer than that between asphalt and rubber.

The contact area directly affects the adhesive friction between the tire and the aggregate. The larger the contact area between coarse aggregates and tires in asphalt pavement per unit area, the more contact points there are between vehicle tires and coarse aggregates. This provides better friction for vehicle movement, indicating a more significant anti-skid capability of the pavement. Therefore, it is necessary to study the influence of aggregate-rubber adhesion friction performance from a microscopic perspective. Wu et al [12] investigated the effect of aggregate morphology on the mechanical properties and permeability of pervious concrete, using sphericity as the index measured by CT and DIP technology. Mechanical and seepage tests were conducted using Avizo software. Dong et al [13] analyzed skid resistance in asphalt pavement, focusing on coarse aggregates. The results showed that micro-texture had a greater impact than angularity, and using high-roughness or anti-wear aggregates enhanced long-term skid resistance. Wang et al [14] utilized the 2nd generation of the Aggregate Imaging Measurement System (AIMS II) and X-ray Computed Tomography (CT) to evaluate the shape of particles and capture the change in their morphological characteristics. Ergin et al [15] produced chip seal samples with varying aggregate types and polishing levels to assess the impact of aggregate microtexture on skid resistance. They used the Micro-Deval (MD) test device to obtain polished aggregates.

Existing research has conducted in-depth studies on the influence of coarse aggregate characteristics on the performance of asphalt mixtures (such as high-temperature stability and low-temperature crack resistance) [16] from the aspects of mechanical properties (polishing value, abrasion value, and crushing value) and morphological characteristics (shape, texture, and angularity) [17], combined with macroscopic experiments and microscopic simulations. Currently, the research on the mechanism of friction between rubber tires and road surfaces mostly adopts macroscopic experiments, and there is less research on the friction between rubber tires and road surfaces at the nanoscale. In order to better understand the skid resistance between tires and road surfaces at the nanoscale, clarify the relationship between tire and road material genes and friction performance, and determine the micro-frictional effect between tires and aggregates, molecular dynamics research methods are used to provide theoretical support for the optimal selection of coarse aggregates based on skid resistance. Yao et al [18] present a comprehensive review of MD simulation of asphalt materials, focusing on modeling, properties, and multi-scale analysis. They discuss asphalt binder and asphalt-aggregate interface models, highlighting differences from real samples and assessing feasibility. Long et al [19] investigated the interface adhesion of nano-silica modified asphalt to aggregate surfaces at the different interfacial situations (aggregate surface irregularity and seawater erosion) using molecular dynamics (MD) simulations. Huang et al [20] constructed the models of asphalt-aggregate interface using four-component molecules of asphalt overlaid on two typical minerals of aggregate, respectively. Xu et al [21] reviews the research status of molecular dynamics simulation of the asphalt-aggregate interface, including modeling, evaluation, influencing factors, and improvement. they discusses asphalt binders and interface models, examining differences with real samples and assessing feasibility.

Therefore, this study used the indentation method to calculate the contact probability ATCR (Aggregate-Tire Contact Ratio) between vehicle tires and aggregates in a unit area of asphalt pavement. Through indoor experiments, the macroscopic adhesive friction coefficient between limestone, basalt, granite, and rubber was tested. These aggregates all came from the same area to ensure that there were no major differences in texture. Furthermore, the main minerals composing the aggregates and the rubber forming the tires were modeled using MS (Material Studio) software [22]. The micro-friction coefficient index AFCAT (Adhesion Friction Coefficient of Aggregate Tire) was proposed. The correlation analysis between the micro-friction coefficient and the macroscopic adhesive friction coefficient was carried out, achieving a microscopic evaluation of the adhesive friction performance between aggregates and rubber.

## 2. Validation test of ATCR for asphalt pavement based on imprint method

### 2.1. Aggregate-tire contact ratio

The Aggregate-Tire Contact Ratio is defined as the probability of contact between coarse aggregates and tires in a unit area of asphalt pavement. It is calculated as the ratio of the contact area between vehicle tires and coarse aggregates to the total area of the mixture, as shown in Eq (1).

$$ATCR=\frac{A_{\alpha }}{A_{\beta }}\times 100\%$$ (1)

**Fig 1:**
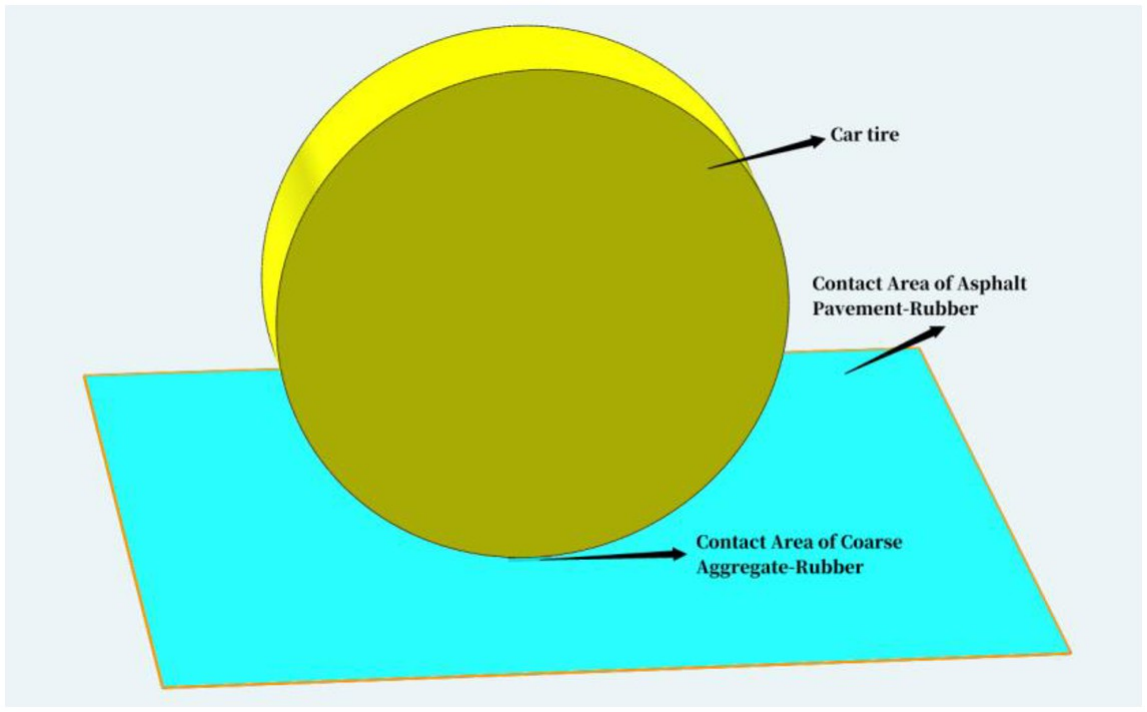
The schematic diagram of ATCR.

Which, ATCR—Contact Probability of Coarse Aggregate-Rubber in Unit Area of Asphalt Pavement (%); *A*_*α*_- Contact Area of Coarse Aggregate-Rubber (mm^2^); *A*_*β*_- Contact Area of Asphalt Pavement-Rubber, mm^2^. The schematic diagram is shown in Fig 1.

### 2.2. Differential verification test process of different graded asphalt pavements

**Fig 2:**
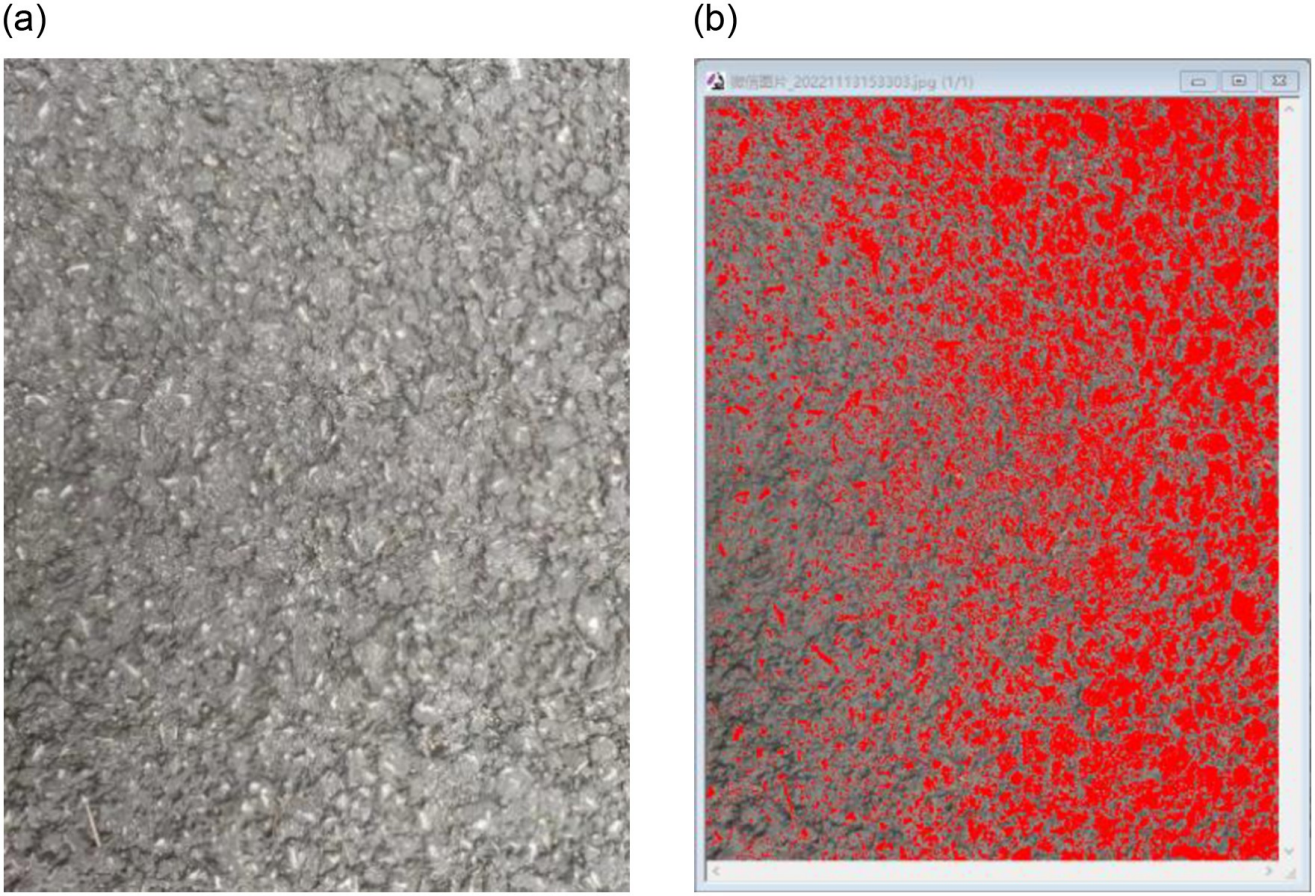
Schematic diagram of asphalt road surface layer. (**a**) Original image. (**b**) Direct processing effect of IPP (Image-Pro Plus).

In order to investigate whether the ATCR values vary among various types of graded asphalt pavements, rutting test specimens featuring AC-13 and SMA-13 median graded formations were employed for the coarse aggregate-rubber contact probability test. Due to the presence of an asphalt film on the surface of freshly formed rutting specimens that was adhered to the coarse aggregate, trichloroethylene was utilized to cleanse the specimen surfaces, thereby exposing the underlying coarse aggregate layer. To ensure the effectiveness of trichloroethylene in cleaning the asphalt, several steps were taken. Firstly, a high-purity grade of trichloroethylene was utilized to minimize potential contaminants. Secondly, the asphalt samples were soaked in trichloroethylene for a sufficient duration, allowing for effective dissolution of any organic contaminants or residues. Additionally, gentle agitation was applied to the samples during the cleaning process to enhance its effectiveness. Following the cleaning procedure, the samples were thoroughly rinsed with distilled water to eliminate any traces of trichloroethylene and subsequently dried completely prior to further analysis. These measures contributed to ensuring the reliability and reproducibility of the results. Traditionally, researchers often capture surface images of the specimens through direct photography, followed by intricate image processing procedures. However, the limited color contrast between asphalt and aggregate leads to heightened computational burden and increased potential for calculation errors, as shown in Fig 2.

To mitigate calculation errors arising from direct road surface measurements and to circumvent the need for complex image segmentation processes, a “printing” technique was employed to determine the contact ratio between road aggregate and tires. The step-by-step procedure was as follows: firstly, a soft brush was utilized to apply a small amount of ink to a specified region on the skid plate; subsequently, an A4 paper was positioned over the inked area, and a rubber sheet was placed on top of the paper, applying a pressure of 0.7 MPa on the contact surface via a weight block. After a duration of one minute, the weight block, rubber sheet, and A4 paper covering the skid plate were removed. A high-definition camera was employed to capture the ink marks on the paper, and the area of ink marks per unit area (representing the contact area between coarse aggregate and tires) was calculated using the IPP software. Combining this with formula 1 enables the determination of ATCR values for various types of graded skid plates.

**Fig 3:**
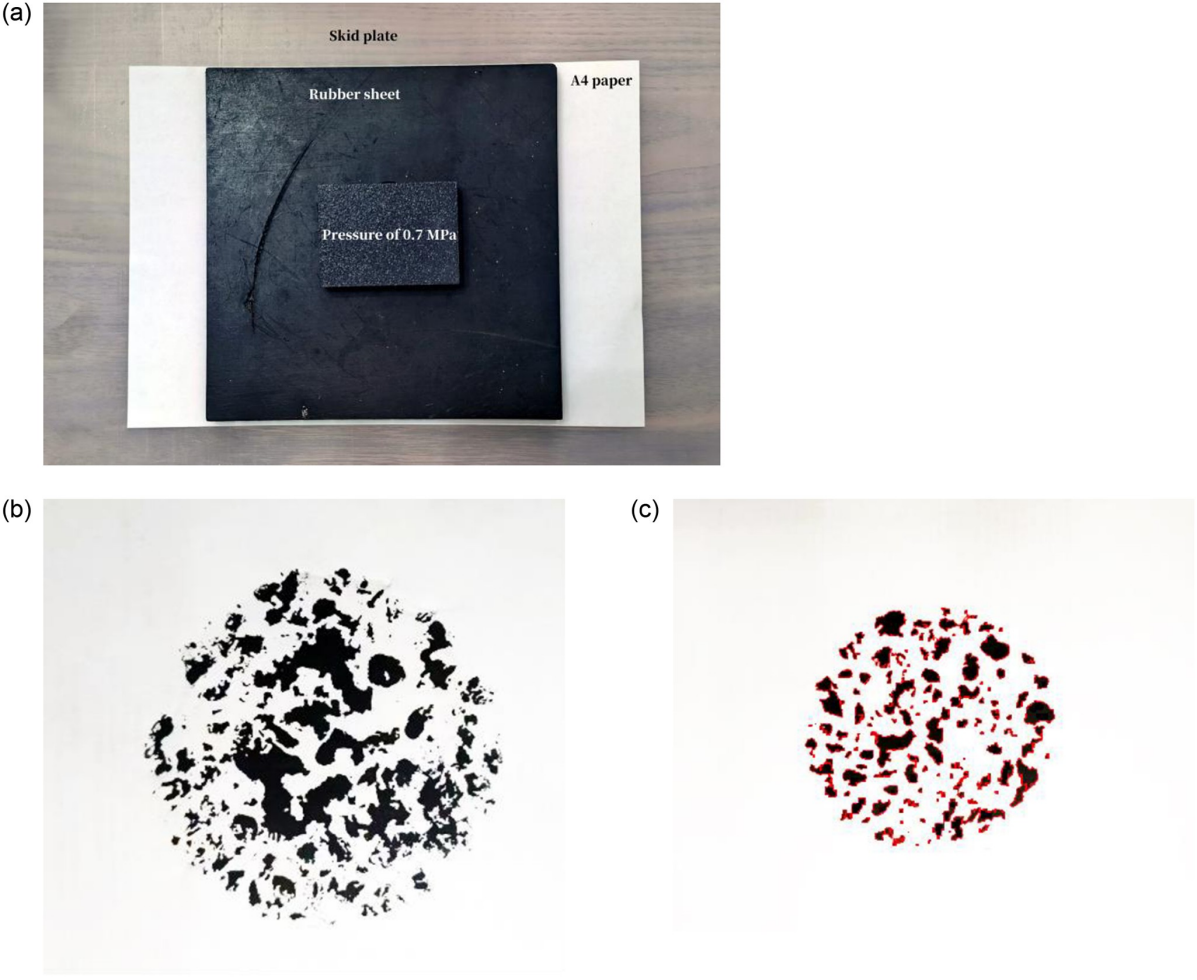
Circular imprint of wheel ruts and processing result. (**a**) Processing procedure. (**b**) Circular imprint of wheel ruts. (**c**) Image processing result using IPP.

In order to ascertain the precision, a coin was positioned onto the A4 paper, and an image was captured using a high-definition camera. The actual area of the coin and the measured area obtained from the print were calculated independently. A comparison between these two values demonstrates a calculation accuracy of up to 98%. The area of the designated region and the printed ink marks were then calculated using the established ruler, and the circular area of the skid plate printing and IPP processing results were shown in Fig 3.

### 2.3. ATCR differences verification test results for different graded asphalt pavements

**Fig 4:**
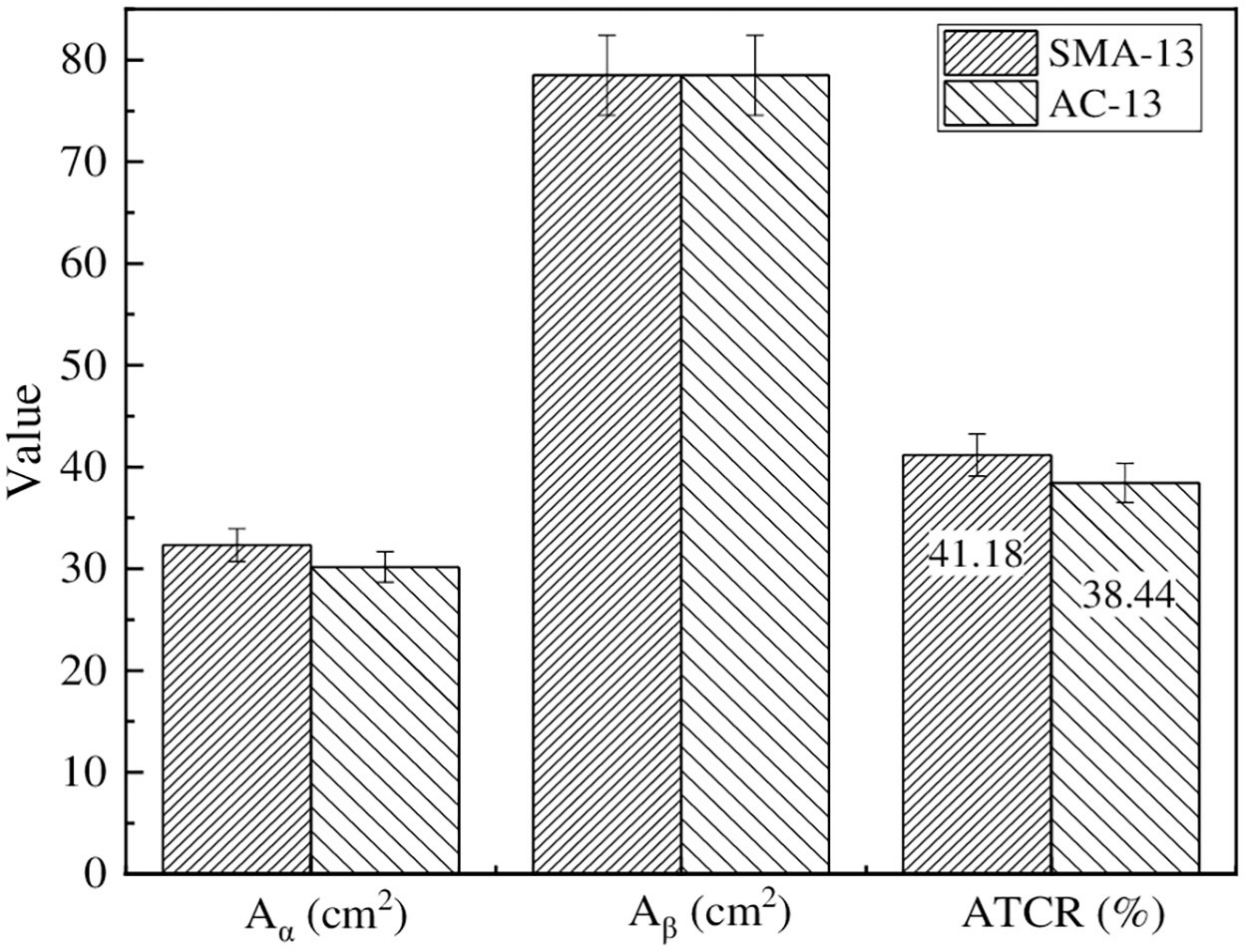
Circular imprint of wheel ruts and processing result.

Fig 4 shows the calculation results of the contact ratio between different graded asphalt pavement aggregates and tires. The graph revealed that there existed discernible variations in the contact ratio between the surface aggregates of different mixture types and vehicle tires, albeit not significantly. Notably, the ATCR value for the SMA-13 graded mixture exceeded that of the AC-13 graded mixture, attributed to the higher proportion of coarse aggregates in the SMA-13 graded mixture. The ATCR values for both graded rutting test specimens were close to 40%, indicating that the contact ratio between tires and pavement aggregates could be determined as 40%. This ratio also demonstrated the significant influence of adhesive friction between pavement aggregates and tires on the skid resistance of the pavement. Therefore, it is necessary to study the adhesive friction between different types of aggregates and the minerals they contain with vehicle tires.

## 3. Indoor test on friction properties of different kinds of aggregate-rubber adhesion

### 3.1. Experimental procedure

In order to quantitatively characterize the adhesive friction performance between rubber and different aggregate types, as well as determine the impact of distinct coarse aggregates on the adhesive friction performance of asphalt pavement, an indoor testing was conducted. Initially, limestone, basalt, and granite slabs were prepared, each with dimensions of 15cm×8cm×3cm. Subsequently, a grinder was employed to polish the corresponding surfaces of the slabs, achieving a smooth surface and reducing the influence of texture and micro-protrusions on the adhesive friction between the slab and rubber. To evaluate the polishing effect, both qualitative and quantitative methods were employed. Visually, the slabs were inspected for uniformity and smoothness. Quantitatively, surface roughness was measured using a profilometer to ensure that the polishing process had achieved the desired surface finish. The necessity for grinding the stone in this study arises from the need to simulate the polishing process that occurs on asphalt pavement surfaces due to traffic wear. In practical pavement applications, while coarse aggregates are not intentionally ground prior to use, they undergo a natural polishing process over time as a result of vehicle tires passing over the surface. This polishing changes the surface texture and mineralogy of the aggregates, which in turn affects their skid resistance performance.

**Fig 5:**
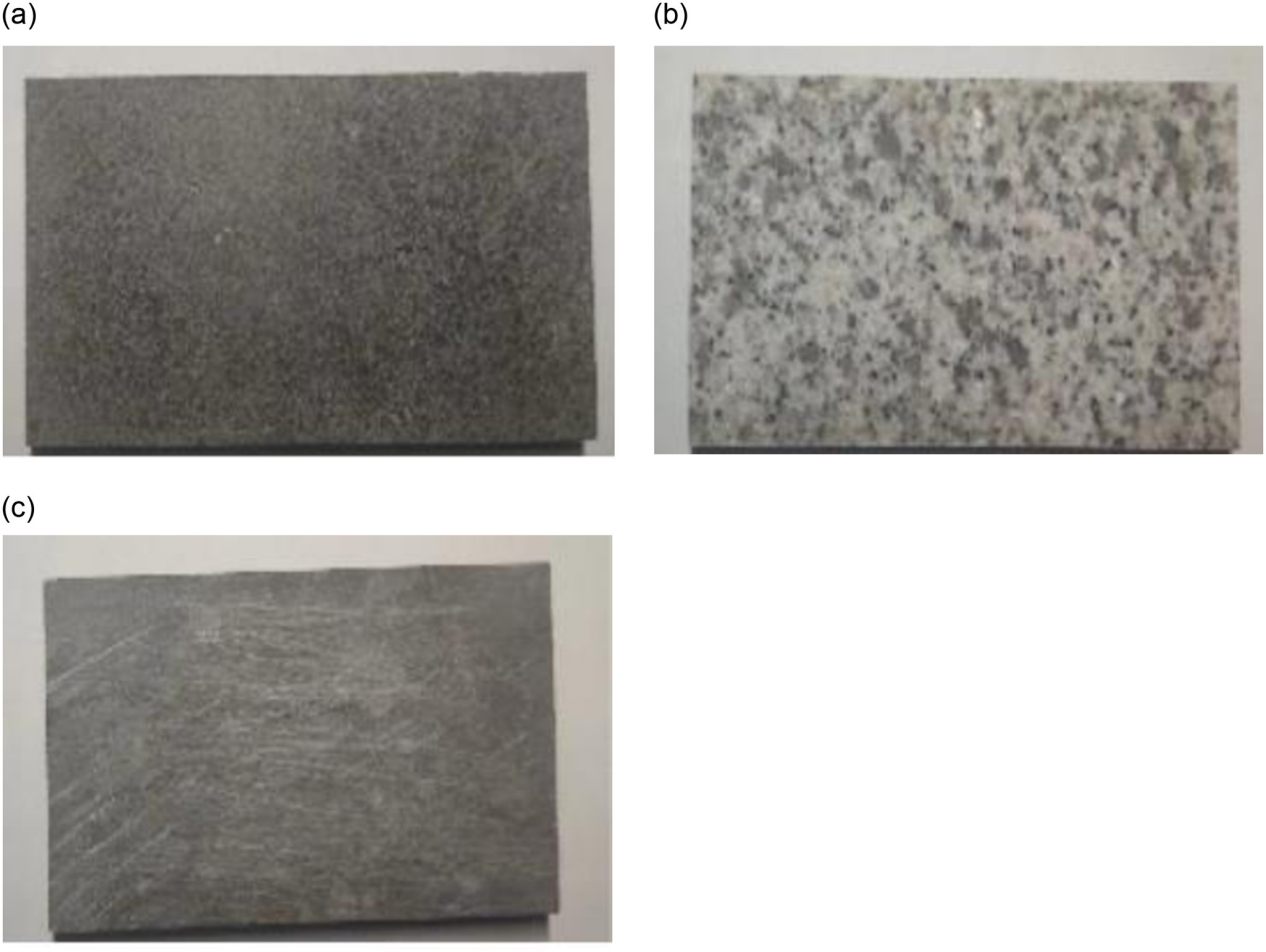
Different types of stone slabs. **(a**) Limestone. (**b**) Basalt. (**c**) Granite.

Fig 5 displays the different types of stone slabs employed in the testing. Finally, a pendulum friction tester was utilized to measure the BPN 20 (British Pendulum Number) value of the limestone, basalt, and granite slab surfaces, providing an evaluation of the adhesive friction between the slabs and tires.

To compare the experimental findings with the computed results from subsequent molecular dynamics, the directly obtained swing values (BPN20) are converted into friction coefficients according to section 2.1.10 of JTG 3450–2019, as described in Eq (2) [23].

$$\mu =\frac{BPN20}{100}$$ (2)

In the above equation, *μ* represents the coefficient of friction, and *BPN*20 represents the measured value obtained from the experiment.

### 3.2. Experimental results analysis

**Fig 6:**
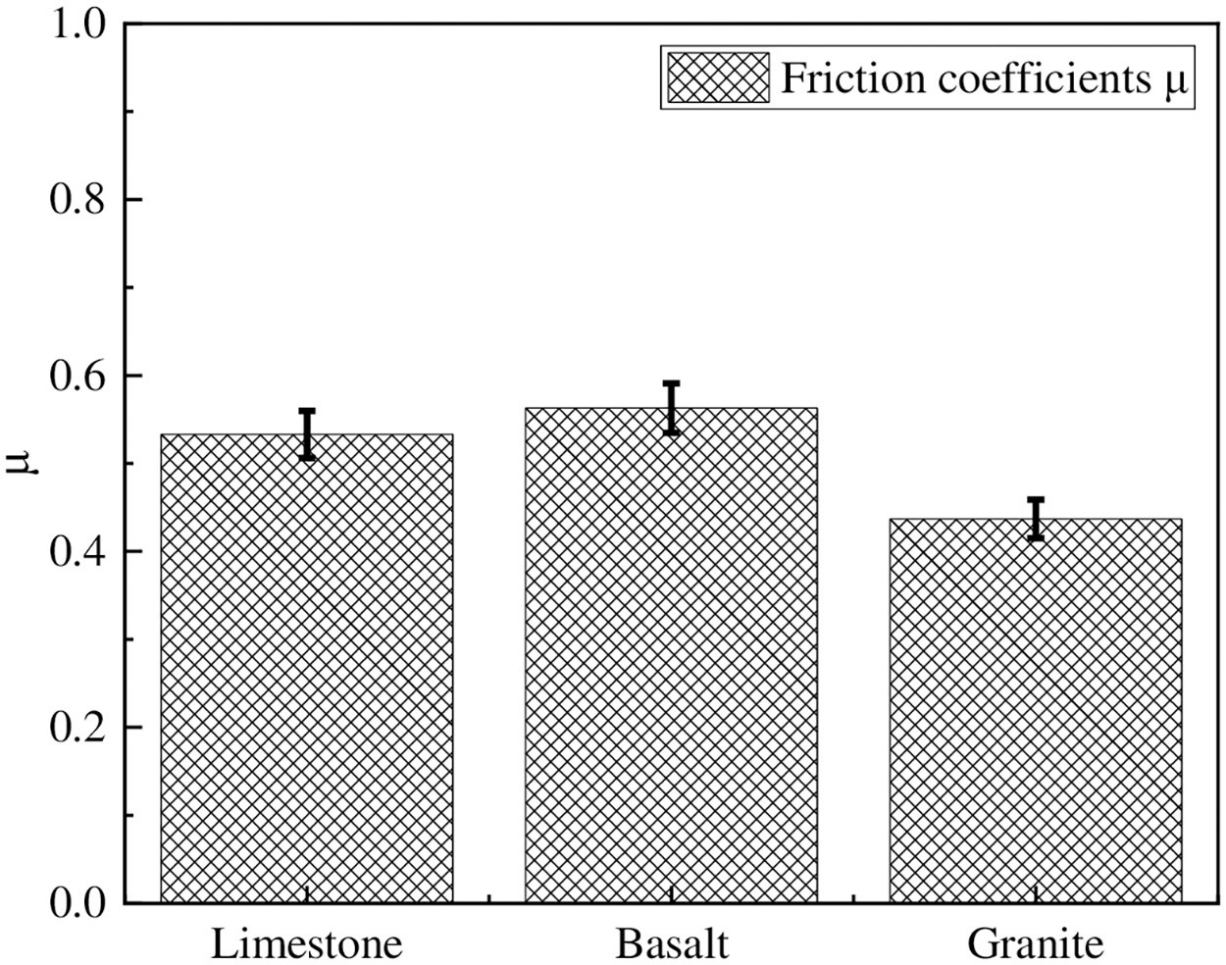
Friction coefficients between different types of stone slabs and rubber.

Fig 6 shows the coefficients of friction between different types of slate and rubber. From the graph, it can be observed that the coefficient of friction is highest between basalt slate and rubber, with a value of 0.56. On the other hand, the coefficient of friction is lowest between granite slate and rubber, measuring only 0.44. There is a significant difference in the coefficient of friction between different types of slate and rubber (*p*<0.05). The reason for this phenomenon may be attributed to the differences in mineral types and content among the three types of slate, leading to varying molecular interactions between different minerals and rubber.

In the context of Fig 6, a parallel test was conducted to evaluate the coefficients of friction between multiple types of slate and rubber. The experimental setup entailed the utilization of a dedicated friction testing apparatus, where a standardized rubber sample was brought into contact with individual slate specimens. The application of a controlled sliding force facilitated the measurement of the frictional force generated between the two materials. This approach allowed for the direct comparison of friction coefficients among different slate types, including basalt slate and granite slate, as evident from the data presented in Fig 6. The significance of this parallel testing methodology lies in its ability to provide quantitative and reproducible results, thereby enhancing the reliability and accuracy of the conclusions drawn regarding the adhesive friction performance between slate materials and rubber.

The above experimental results provide preliminary information on the adhesive friction performance between different types of coarse aggregates and stone materials and rubber. However, indoor macroscopic experiments have certain limitations, as they cannot guarantee a completely smooth surface of the stone slab, which may affect the test results due to the surface texture of the stone. Additionally, the main mineral composition of coarse aggregates directly affects their performance. Therefore, molecular dynamics simulations are used to study the adhesive friction performance between different minerals (material genes) and rubber. This approach allows for a microscopic explanation of the adhesive friction between aggregates and rubber and further validates the reliability of indoor macroscopic experiments.

## 4. Construction of different aggregate mineral components and rubber molecular models

### 4.1. Molecular dynamics calculation parameters

A force field is an empirical potential function used to describe the interactions between particles in a system. It is a key component of molecular simulation calculations, describing the local environment of a particle and the interactions between it and surrounding particles [24]. The ensemble originates from statistical mechanics and can be defined as a set of systems under certain conditions. Molecular dynamics simulations mainly involve four ensembles: microcanonical ensemble, canonical ensemble, isothermal-isobaric ensemble, and isenthalpic-isobaric ensemble [25]. In this study, the canonical ensemble (NVT) and isothermal-isobaric ensemble (NPT) are used for the molecular dynamics simulations.

The canonical ensemble (NVT) represents a system with a fixed number of particles (N), volume (V), and temperature (T). It is a typical representation of systems simulated using Monte Carlo methods. The isothermal-isobaric ensemble (NPT) represents a system with a fixed number of particles (N), pressure (P), and temperature (T). Therefore, the implementation of this ensemble requires the use of a thermostat and barostat to maintain the system at constant temperature and pressure.

### 4.2. Selection of aggregate minerals and rubber molecules

#### 4.2.1. Selection of typical rubber molecules

Tires are complex structures composed of rubber, steel wires, nylon threads, additives, and other components. The rubber used in car and truck tires is mainly natural rubber (NR), butadiene rubber (BR), and styrene-butadiene rubber (SBR) [26]. The primary focus of this study centers on investigating the frictional interaction between rubber and various types of minerals. To maintain control over variables, a single type of rubber is employed as a substitute for the rubber tire. Consequently, natural rubber has been selected as the subject of investigation.

**Fig 7:**
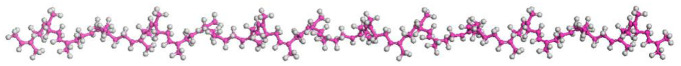
NR (natural rubber) molecular chain.

Natural rubber (NR) is formed by the polymerization of isoprene monomers. The degree of polymerization (number of repeating monomers) has a significant impact on the results of molecular simulations. Previous studies have found that the minimum degree of polymerization for NR is 20 [27]. To ensure the reliability of the simulation results, a degree of polymerization of 30 is chosen for NR in this study. The molecular chain of NR is shown in Fig 7.

#### 4.2.2. Typical selection of coarse aggregate mineral molecules

**Fig 8:**
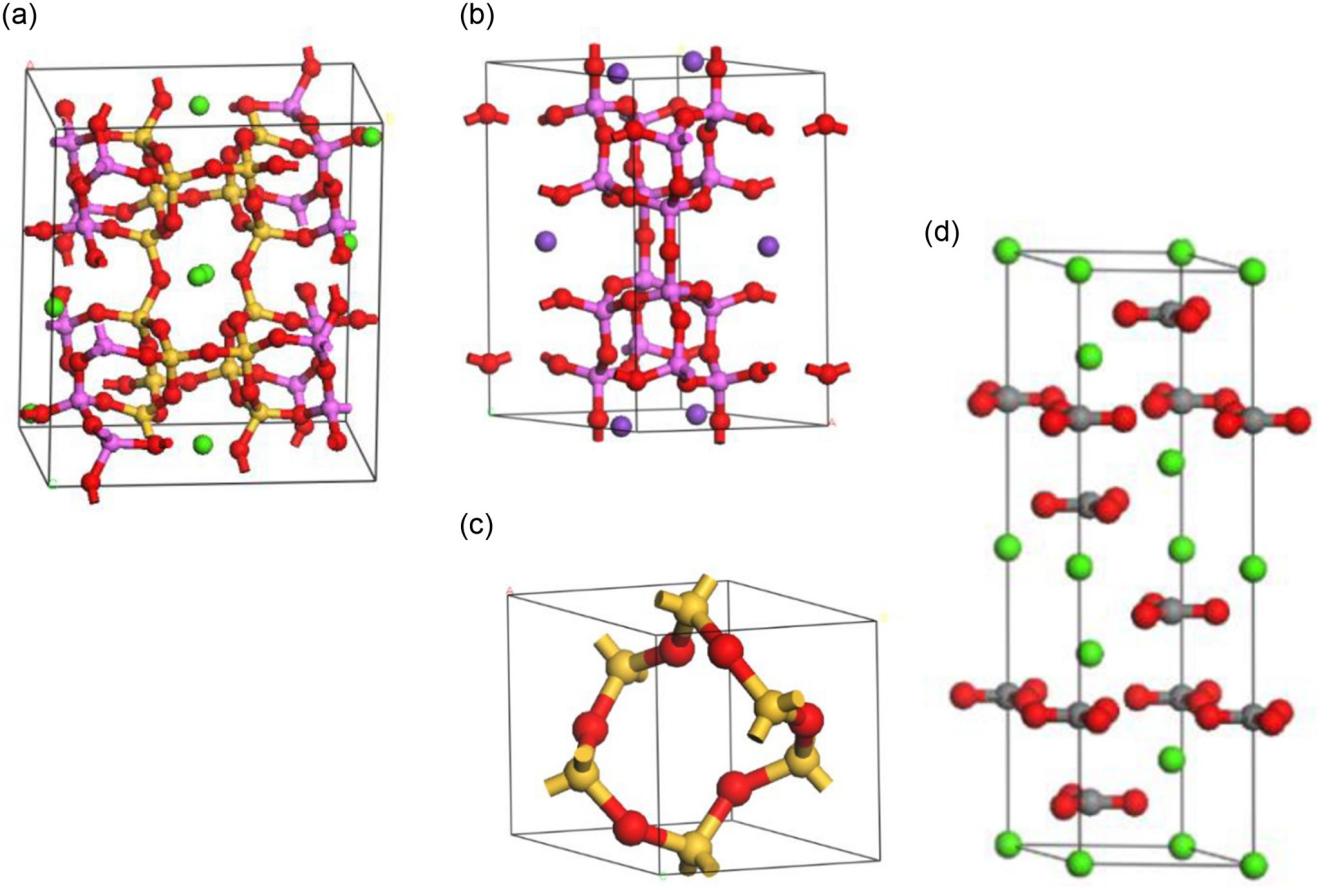
Models of mineral crystals. (**a**) Sodium feldspar. (**b**) Orthoclase feldspar. (**c**) Quartz. (**d**) Calcite.

Aggregates typically comprise various minerals, and the composition and quantity of different minerals exert a notable influence on the physical, chemical, and mechanical properties of these aggregates. For the purpose of this study, limestone, basalt, and granite were chosen as the aggregate types. The primary mineral associated with limestone is calcite. Basalt typically consists of minerals such as sodium feldspar and orthoclase feldspar. Granite is primarily composed of quartz. Accordingly, the molecular models of these four minerals were incorporated into the molecular dynamics simulation software. Fig 8 shows the crystal models of these minerals.

### 4.3. Construction of mineral and rubber molecular constrained shear models

#### 4.3.1. Construction of NR molecular model

**Fig 9:**
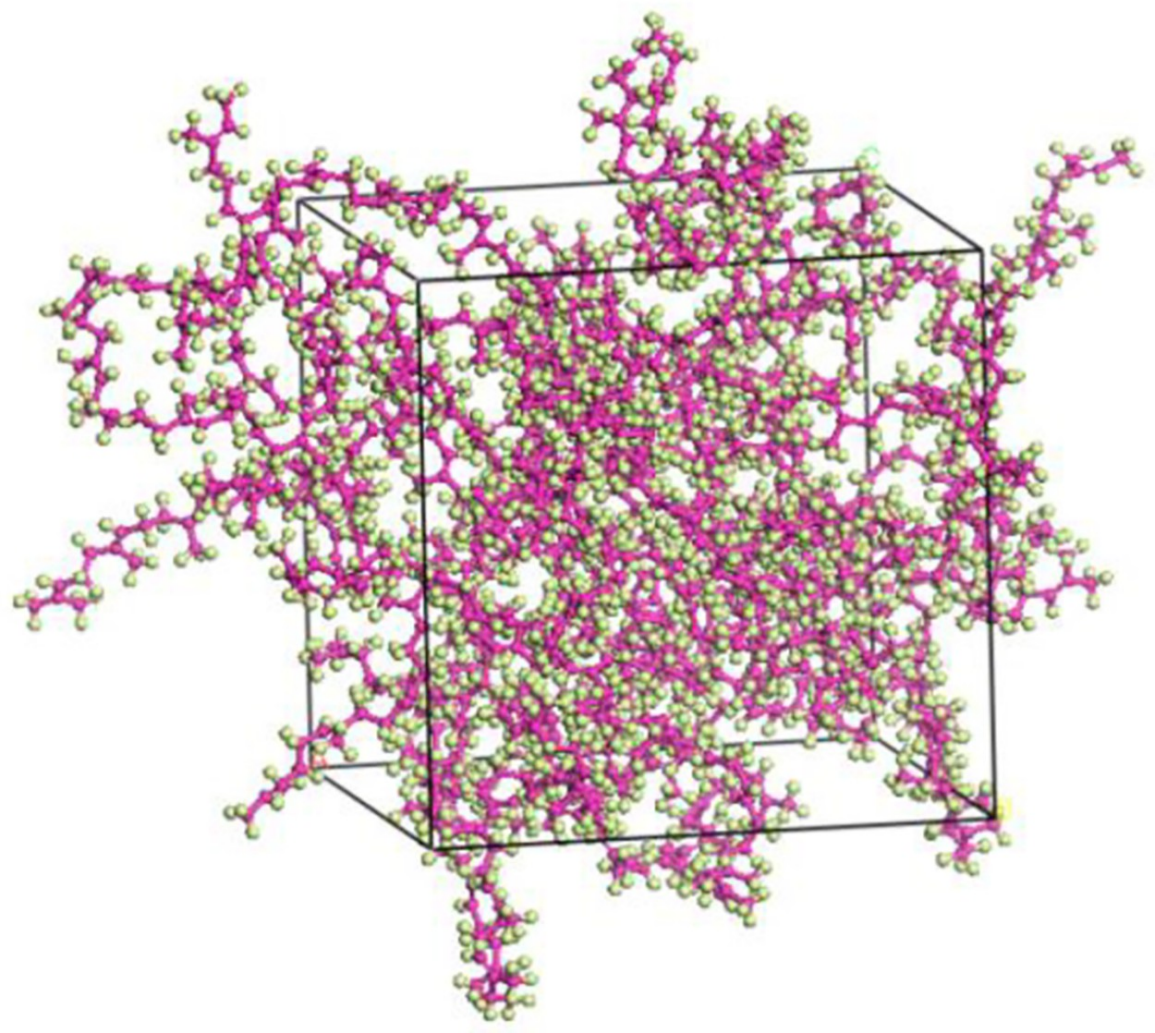
Initial crystal cell model of NR molecule.

Utilizing the MS software, we constructed the NR molecule and the four mineral molecule unit cells mentioned above. These components were then combined to generate a constrained shear structure model for dynamic calculations. The NR molecule chains consist of 30 monomers of isoprene, connected head-to-tail to construct an amorphous unit cell. A density of 0.6 g/cm^3^ was assigned to the initial unit cell model of NR, and periodic boundary conditions were applied. The resulting initial unit cell model of NR possesses dimensions of 32.39 x 32.39 x 32.39Å3. Fig 9 displays the depiction of the initial unit cell model.

**Fig 10:**
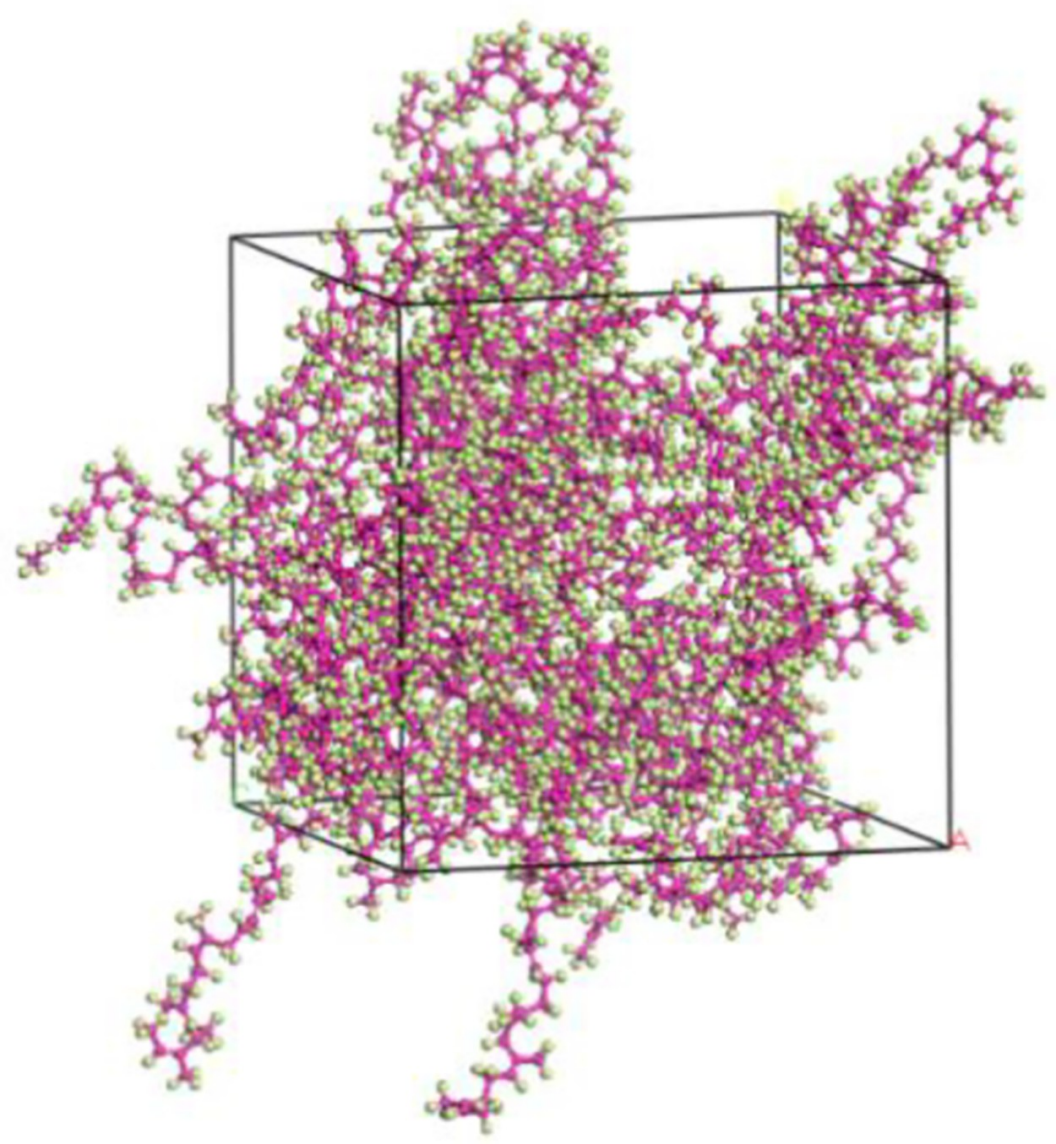
NR crystal cell model after geometric optimization.

The initial unit cell model may have poor molecular structures, molecular overlap, and stacking phenomena. To ensure a reasonable distribution of NR molecule chains in the unit cell model, the Geometry Optimization function in the Forcite module is used to perform geometric optimization on the initial unit cell model of NR, minimizing the energy of the model. The specific parameter settings are as follows: using the smart algorithm, performing 10,000 iterations of optimization, selecting fine precision, setting a cutoff radius of 15.5Å, using the COMPASS II force field, and solving electrostatic non-bonded interactions and van der Waals non-bonded interactions using the Ewald and Atom Based methods, respectively. The charges are set to Forcefield Assigned, and the software is run. After geometric optimization, the NR unit cell model should reach a stable and convergent energy state. The optimized unit cell model is shown in Fig 10.

**Fig 11:**
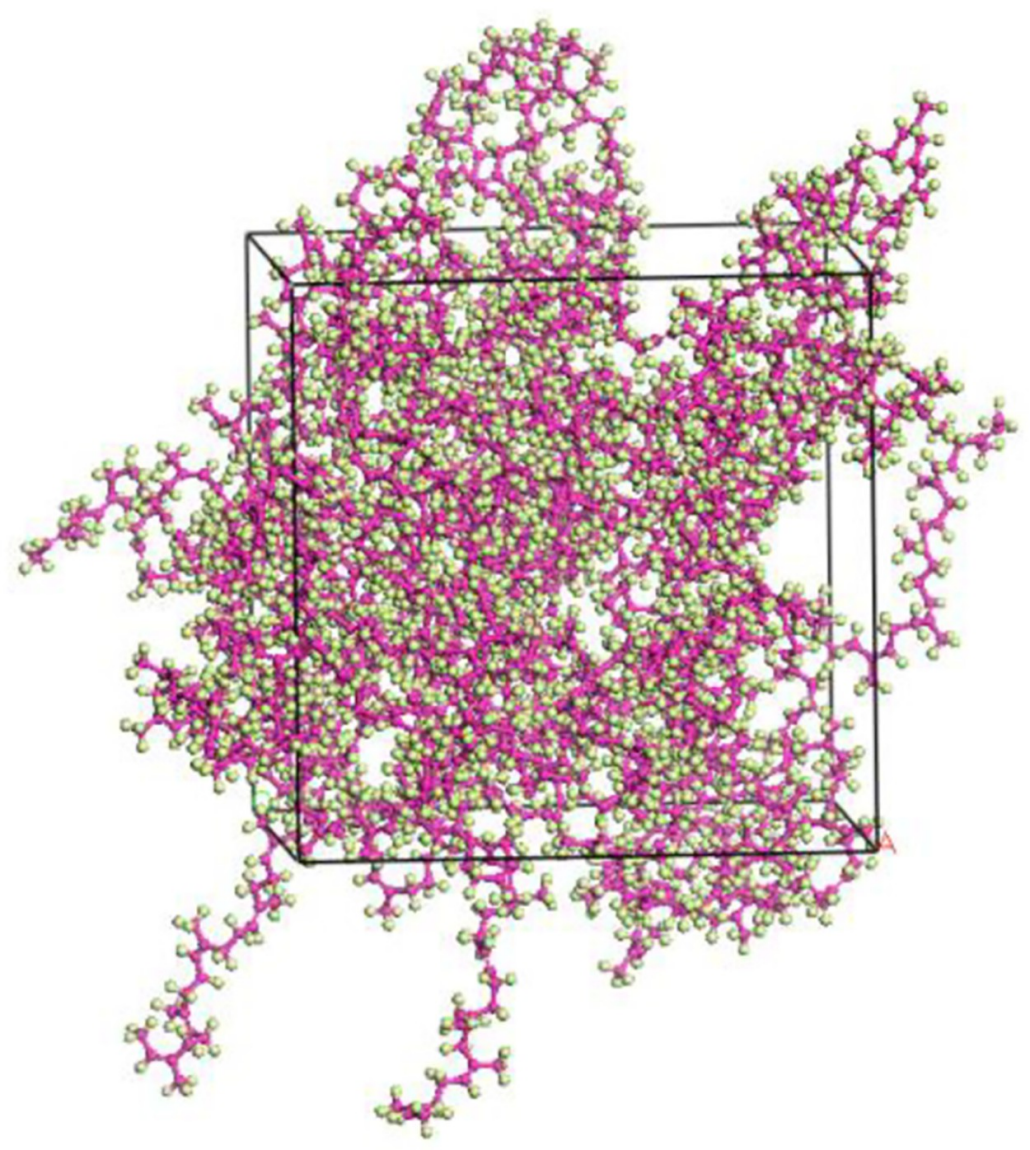
NR model after annealing.

**Fig 12:**
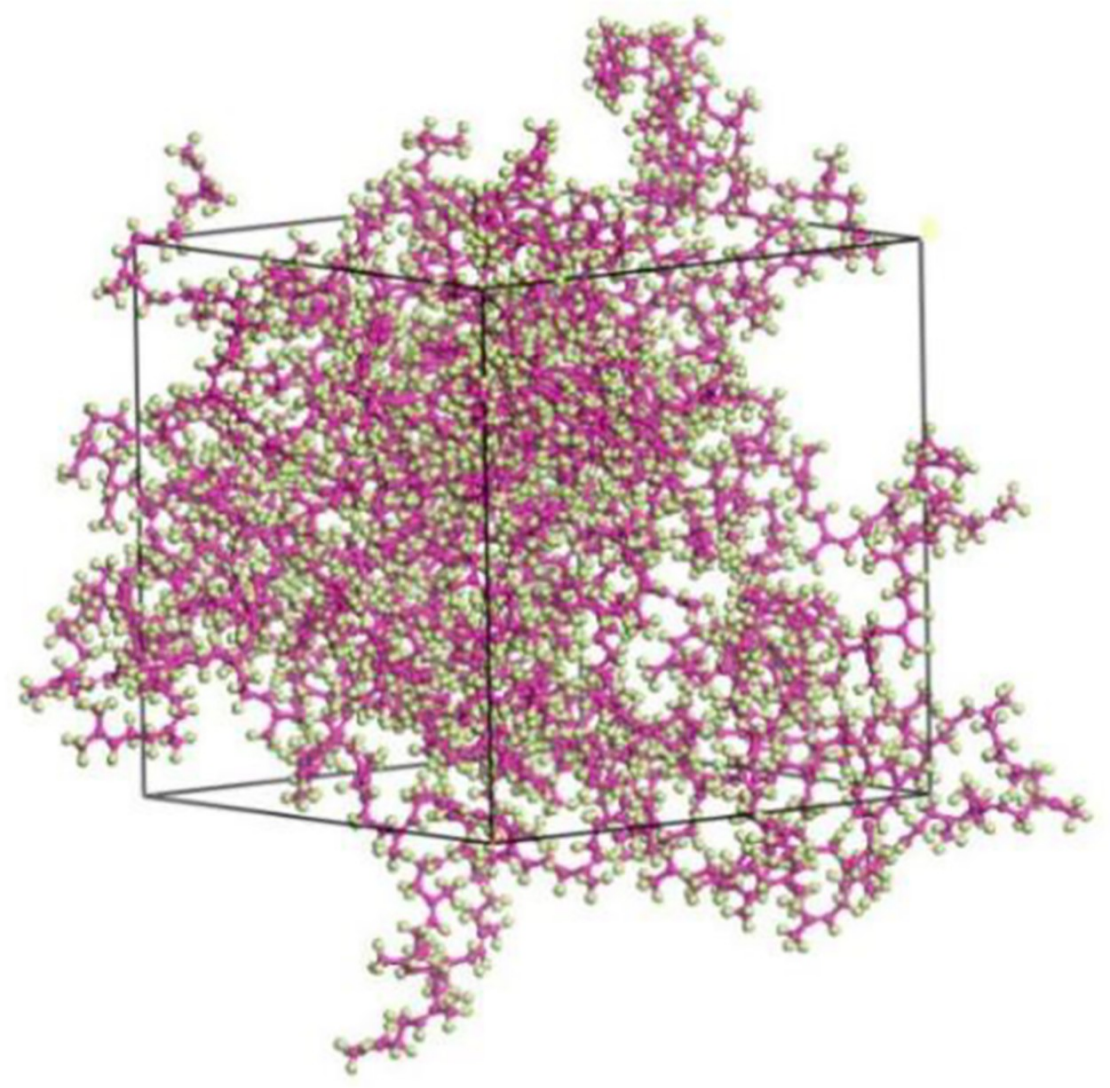
Final NR crystal cell model.

After completing the geometric optimization of the NR unit cell model, in order to further stabilize its structure and reduce its energy, annealing treatment is performed. Annealing treatment allows the model to fully expose its internal structure in a high-temperature environment, further eliminating unreasonable structural arrangements in the model, and providing a more balanced geometric conformation for subsequent dynamic calculations to overcome energy barriers. After annealing treatment, the structure with the lowest total energy in the process file is selected for dynamic simulation calculations, making the constructed NR unit cell model closer to the actual state. The parameters related to annealing treatment and dynamic simulation calculations are shown in Tables 1 and 2, respectively. The annealing model structure and the final NR unit cell model are shown in Figs 11 and 12, respectively.

**Table 1:** Parameters related to annealing treatment.

Parameter	Parameter setting value
Period	5
Temperature (K)	250–500
Ensemble	NVT
Cutoff distance (Å)	15.5
Time (ps)	50ps

**Table 2:** Parameters for dynamic calculations.

Parameter	Parameter setting value
Temperature(k)	298
Pressure (atm)	1atm
Ensemble	NPT
Accuracy	fine
Time (ps)	200
Force field	COMPASS II

#### 4.3.2. Construction of mineral crystal lattice models

**Fig 13:**
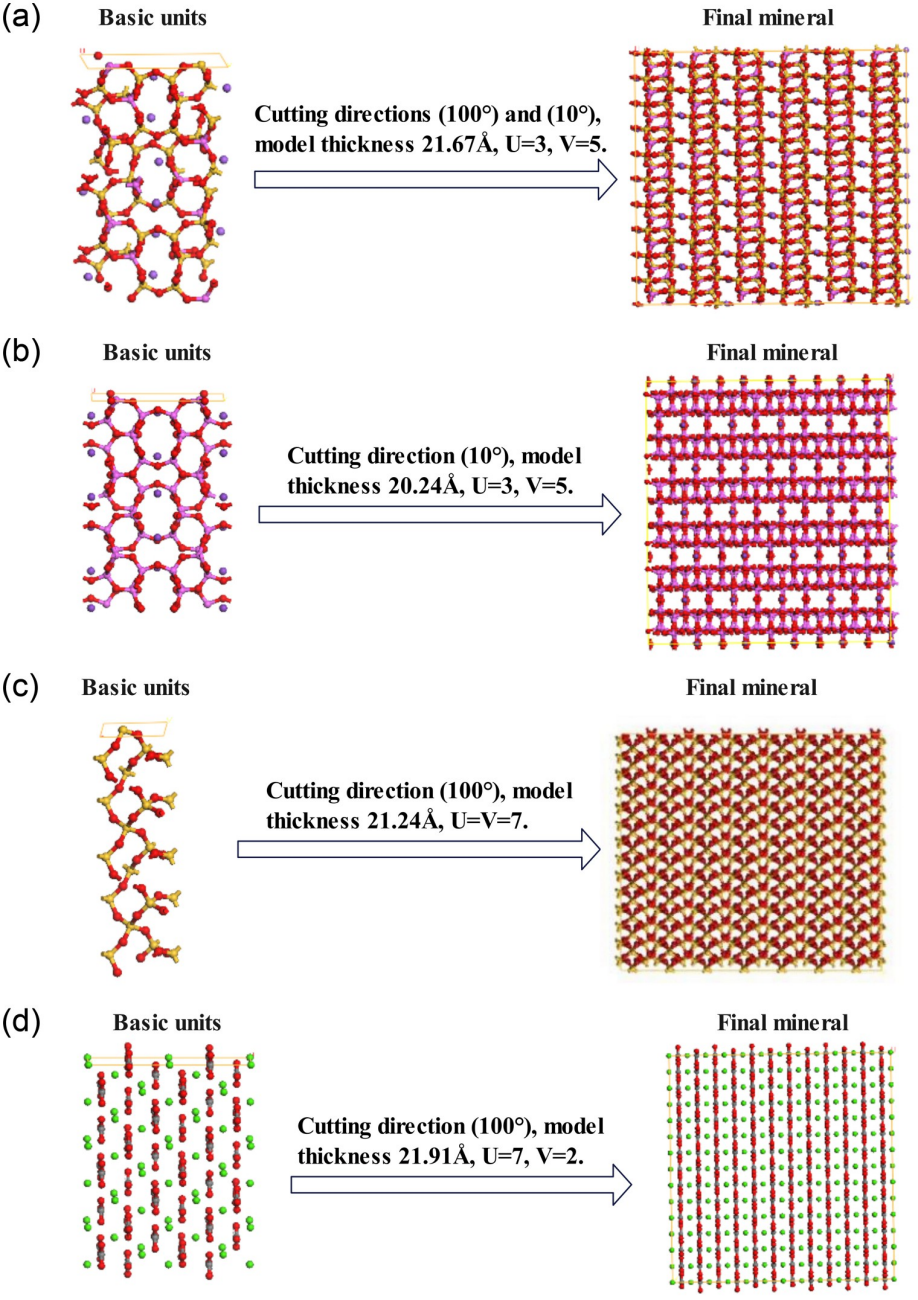
Crystal cell models of four minerals. (a) Sodium feldspar. (b) Orthoclase feldspar. (c) Quartz. (d) Calcite.

In order to examine the adhesive friction interaction between rubber and various mineral types, four mineral structures were obtained from the MS structure database and subsequently modeled. To expose the pertinent surfaces, the minerals were cut along the 100° direction. It has been found through several tests that cutting along the 100° direction allows for full exposure of the contact surface. As the crystal cell models of plagioclase feldspar and alkali feldspar lacked a 90° angle, two cuts were required [28]. Subsequently, the Supercell function was utilized to construct mineral crystal cell models that aligned with the area of the rubber crystal cell. The specific parameters employed in these models are detailed in Fig 13. As can be seen in Fig 13, U is the length of the cell and V is the width of the cell.

**Fig 14:**
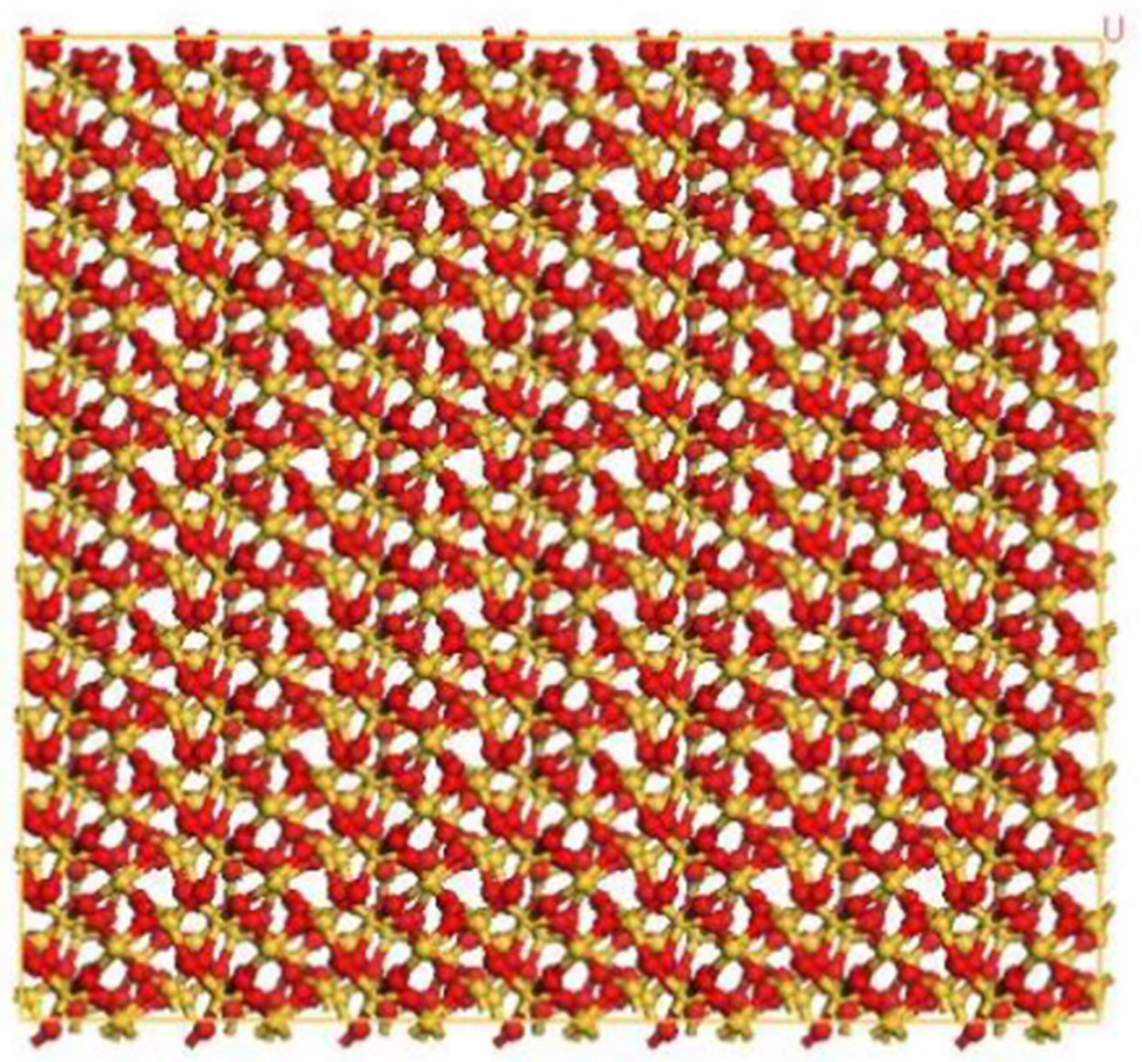
Quartz crystal cell model after geometric optimization.

After applying the processing method used for the NR crystal cell model, the initial step involved performing geometric optimization to minimize the energy of the crystal cell model. Through this optimization process, the energy of the mineral crystal cell model was expected to reach a stable and convergent state. As an example, Fig 14 depicts the optimized crystal cell model, specifically using quartz.

#### 4.3.3. Construction of aggregate mineral and rubber molecular confinement shear model

**Fig 15:**
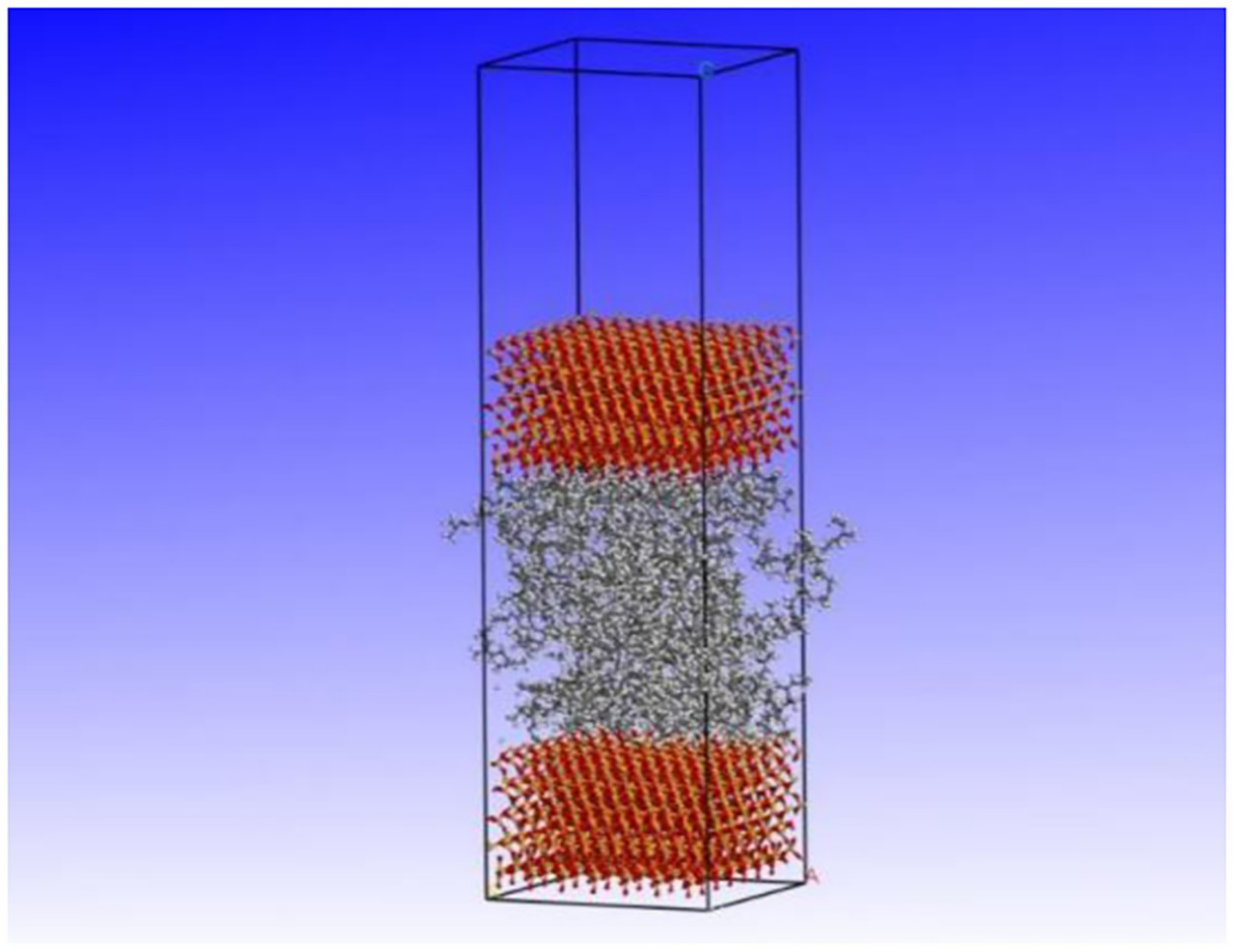
Equilibrium state three-layer molecular crystal cell model.

After completing the establishment and optimization of the NR and mineral crystal cell models, use the “Build Layers” function to combine the NR and mineral crystal cell models, constructing a three-layer molecular crystal cell model in a “sandwich” style, with the mineral crystal cell model as the upper and lower layers and the NR crystal cell model as the core. This configuration allows for a direct interaction between the mineral and rubber layers, simulating the adhesive friction forces that arise during tire-road contact. By constraining the shear movement of the model, it enables the measurement of adhesive friction coefficients at the micro-scale level. Design a 40Å thick vacuum layer to eliminate the influence of periodic boundary conditions. Perform 5000 iterations of geometric optimization on the constructed three-layer molecular crystal cell model, followed by a 200ps dynamic optimization (under the NVT ensemble) on the geometrically optimized model. This process aims to achieve the lowest energy equilibrium state for the model. The final constrained shear model is shown in Fig 15.

The rationale behind using the sandwich model in this study was to provide a more accurate and representative evaluation of the adhesive friction performance between aggregate minerals and rubber at the micro-scale level. This model was specifically designed to mimic the actual contact conditions that occur between the tire and the aggregate particles on the road surface. The use of this model reflects field conditions in several ways. Firstly, it takes into account the heterogeneity of the road surface, which consists of various types of aggregate minerals with different physical and chemical properties. The sandwich model allows for the evaluation of specific mineral-rubber interactions, providing insights into how these properties affect skid resistance. Secondly, the model incorporates the dynamic nature of tire-road contact. In real-world conditions, tires constantly move over the road surface, experiencing friction forces that depend on the adhesive properties of the materials in contact. The sandwich model simulates this dynamic interaction by evaluating the adhesive friction coefficients under shear forces, which are representative of the forces acting on a tire as it rolls over the road. Finally, the results obtained from the sandwich model can be correlated with macroscopic friction measurements, as demonstrated in the study. This correlation provides validation for the model’s ability to accurately represent field conditions and enables a more comprehensive understanding of the factors influencing skid resistance performance.

### 4.4. Validation of the rationality of the molecular crystal cell model

In order to assess the reliability of the constructed NR crystal cell model, a comparison was made using the solubility parameters and density metrics of the model with experimental results [29]. This comparison aimed to evaluate the rationality of the molecular model construction. The NR crystal cell model was evaluated using the Cohesive Energy Density function within the Forcite module of the MS software. The calculated solubility parameter for the NR crystal cell model was determined to be 17.34 J/cm^3^, while the density was found to be 0.88g/cm^3^. It was noted that the reported true solubility parameter for NR is 17.00 J/cm^3^, with a corresponding density of 0.91 g/cm^3^ [30, 31]. The simulated solubility parameter exhibited a mean square error of 5.7%, while the density demonstrated a mean square error of 3.4%, both of which fell within 6% of the true values. Consequently, the established NR crystal cell model proved suitable for studying the friction characteristics between rubber and minerals as a substitute for real natural rubber.

## 5. Analysis of adhesive friction between mineral components and rubber based on molecular simulation

### 5.1. Dynamic calculation process

**Fig 16:**
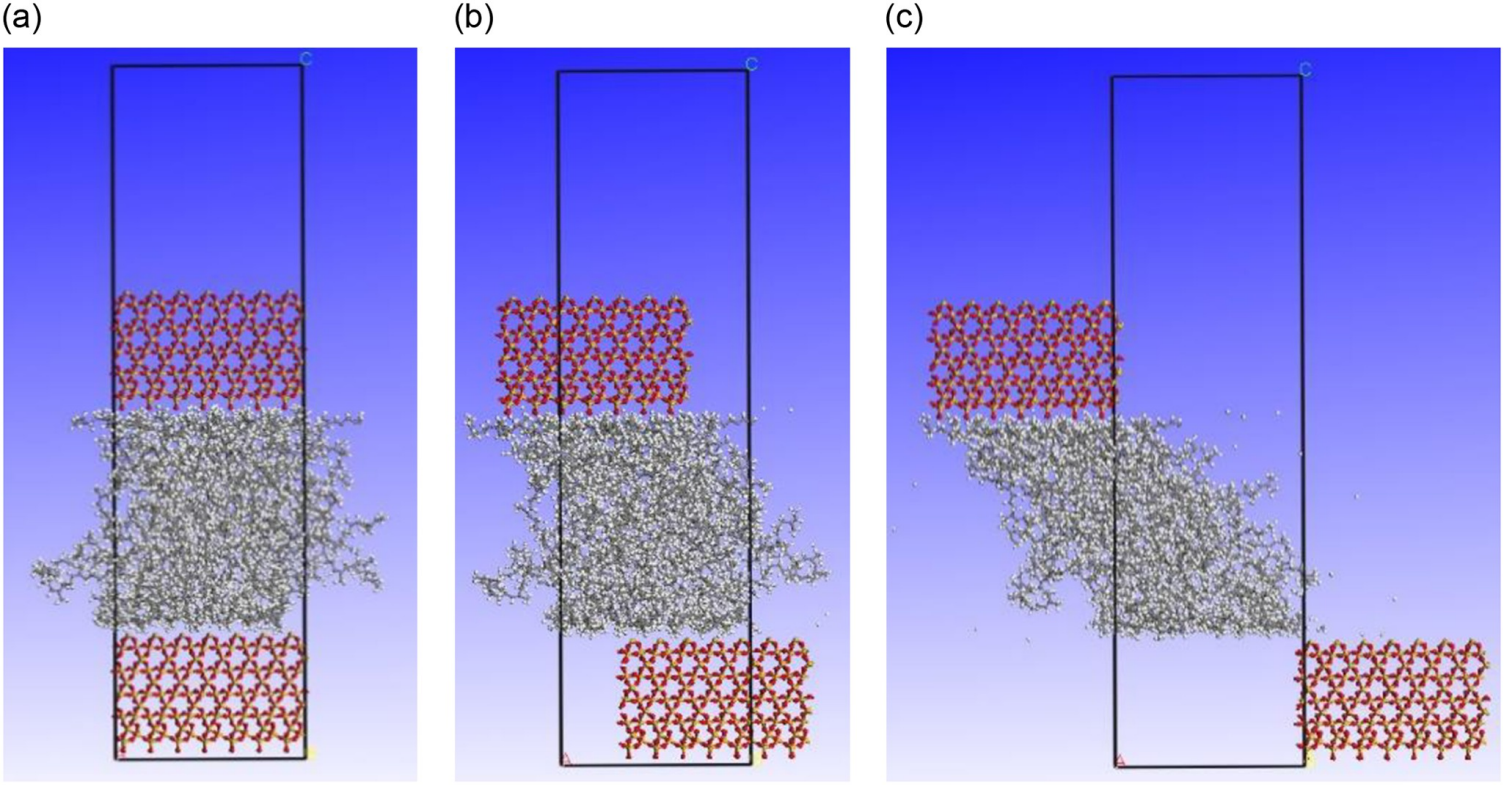
Quartz-NR-quartz model constrained shear process. **(a)** Cutting starts. **(b)** Cutting in progress. **(c)** Cutting ends.

After constructing, optimizing the geometry, and dynamically optimizing the mineral-rubber-mineral three-layer model, the constrained shear of the mineral-rubber-mineral three-layer model was performed to simulate the interaction between the tire and the exposed aggregate on the road surface. The shear load was applied to the mineral molecular models located at the top and bottom, causing relative sliding between the upper and lower layers of mineral molecular models and the middle layer of NR molecular models at a fixed velocity. By studying the interaction forces between mineral and NR molecules, the frictional effects between the two types of molecules can be determined. The constrained shear process was conducted under the NVT ensemble, with an environmental temperature of 298 K and a velocity set at 0.17 Å/ps (macroscopic conversion speed of 60 km/h). The schematic diagram of the changes in the three-layer molecular crystal cell model during the constrained shear process is shown in Fig 16 (using quartz as an example).

The friction coefficient of the contact surface between the NR molecular model and the mineral molecular model during the constrained shear process is calculated as shown in Eq (3) [32].

$$\mu =\frac{F}{N}=\frac{S_{x}}{S_{z}}$$ (3)

Which, *μ* is the friction coefficient; *F* is the longitudinal force at the tire-road contact interface; *N* is the vertical stress at the tire-road contact interface; *S*_*x*_ is the longitudinal average stress of the sliding layer; and *S*_*z*_ is the average vertical stress of the sliding layer.

### 5.2. Friction analysis between different minerals and rubber at the same cutting speed

**Fig 17:**
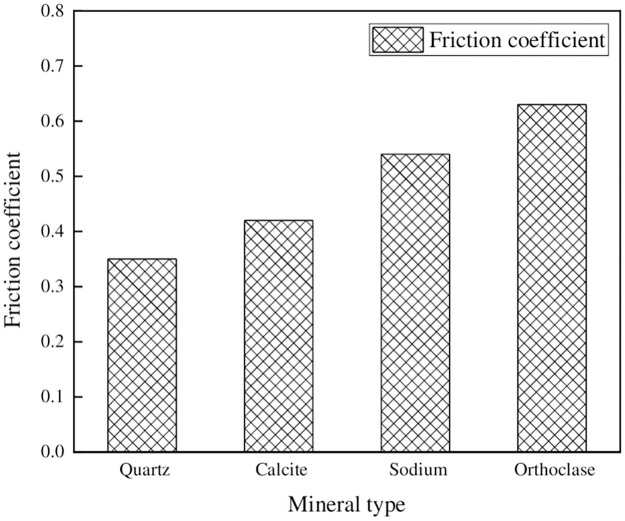
Friction coefficient at a shear rate of 0.17Å/Ps.

The calculated friction coefficient between the mineral cell and the rubber cell at a shear rate of 0.17Å/ps is shown in Fig 17.

From the graph, it could be observed that there were significant differences in the friction coefficients between different types of minerals and natural rubber at the same shear rate, with Orthoclase>Sodium>Calcite>Quartz. When considering only the friction between minerals and rubber for coarse aggregate selection, it was preferable to choose aggregates with higher feldspar content and avoid excessive quartz content in the aggregates.

The reason for this phenomenon may be that the van der Waals force between quartz and rubber was smaller than that between feldspar and rubber, resulting in a higher friction coefficient between feldspar and rubber compared to quartz and rubber. Gao et al. [33] found that the interaction energy between feldspar and asphalt was much greater than the interaction energy between calcite and asphalt caused by strong electrostatic forces, suggesting a similar interaction may exist in the aggregate-rubber system. To further explain the frictional interaction between different minerals and natural rubber, the calculation results of total energy, potential energy, kinetic energy, and other energy parameters for each model were obtained from the output. Combined with the dynamic interface simulation, the potential energy change of the system before and after the constrained shear operation of the rubber-aggregate interface model was considered. The evaluation index *Wτ* was proposed to compare and analyze the differences in the energy required for the constrained shear simulation of each aggregate mineral model, as shown in Eqs (4) and (5).

$$\Delta E=E_{f}-E_{i}$$ (4)

$$W_{\tau }=\frac{\Delta E\times \eta }{2S}$$ (5)

**Fig 18:**
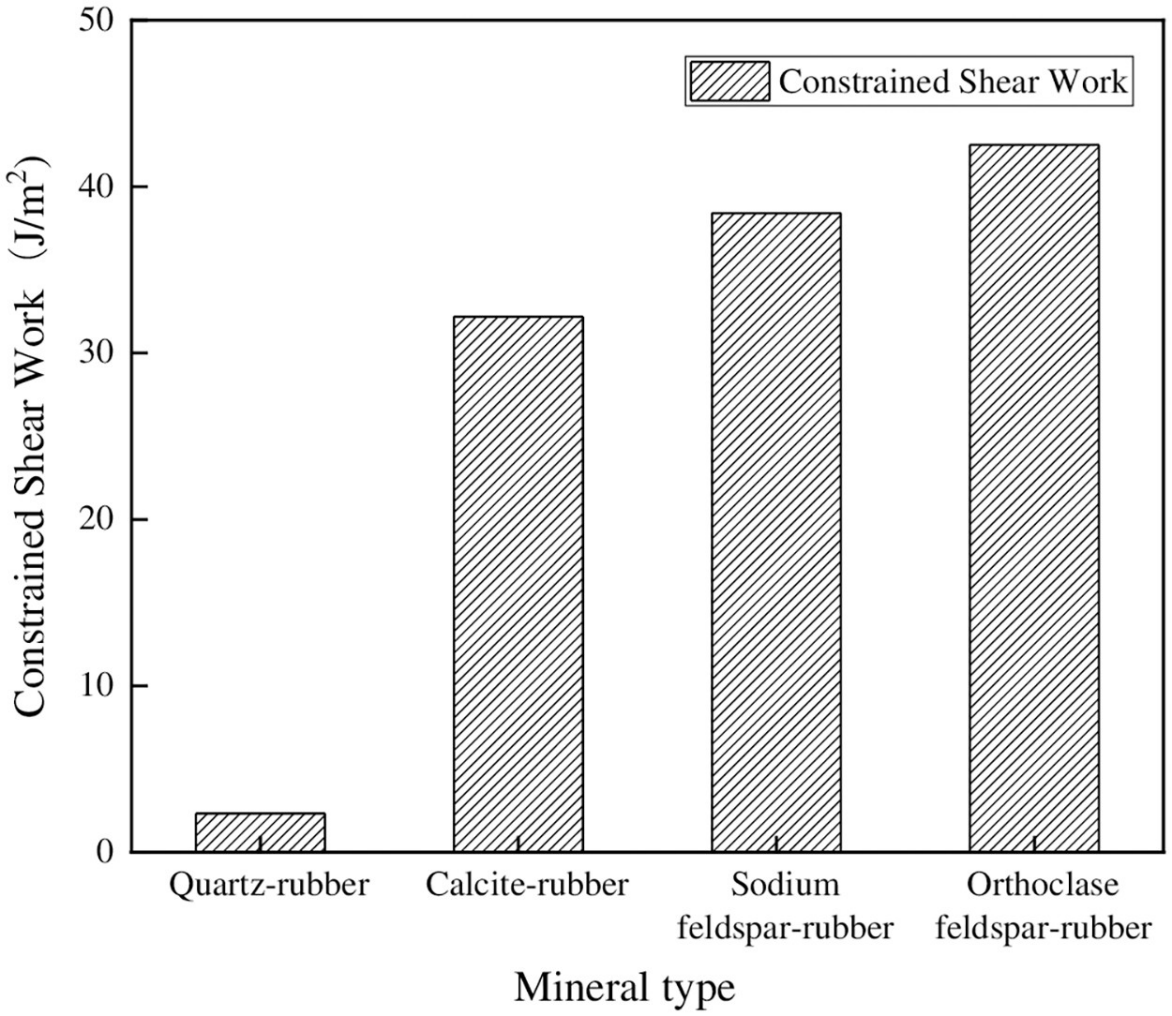
Constrained shear work between minerals and rubber.

In this equation, *ΔE* represents the potential energy difference before and after the model calculation. The terms *E*_*f*_(kcal/mol) and *E*_*i*_(kcal/mol) respectively denote the total potential energy after and before the calculation of the rubber-filler constrained shear model system. *Wτ*(J/m^2^) represents the constrained shear work, *η* is the energy conversion coefficient, and in this case, it is taken as 6.95 × 10^−21^. *S* (m^2^) represents the contact area between the filler and rubber model interface. For comparative analysis, the calculated results of *Wτ* for each model control group were plotted in the same bar chart, as shown in Fig 18.

It was evident that significant disparities existed in the constrained shear work of various minerals when in contact with natural rubber. More specifically, the constrained shear work observed between quartz and natural rubber was the smallest, measuring at 2.34 J/m^2^, while the largest constrained shear work was exhibited between feldspar and rubber, amounting to 42.5 J/m^2^. Notably, the observed trend in the variation of mineral-rubber constrained shear work aligned with the concurrent trend in the mineral-rubber friction coefficient. Consequently, it is reasonable to conclude that the mineral-rubber constrained shear work can elucidate the friction coefficient between these two materials.

### 5.3. Friction analysis between different minerals and rubber at different shear rates

**Fig 19:**
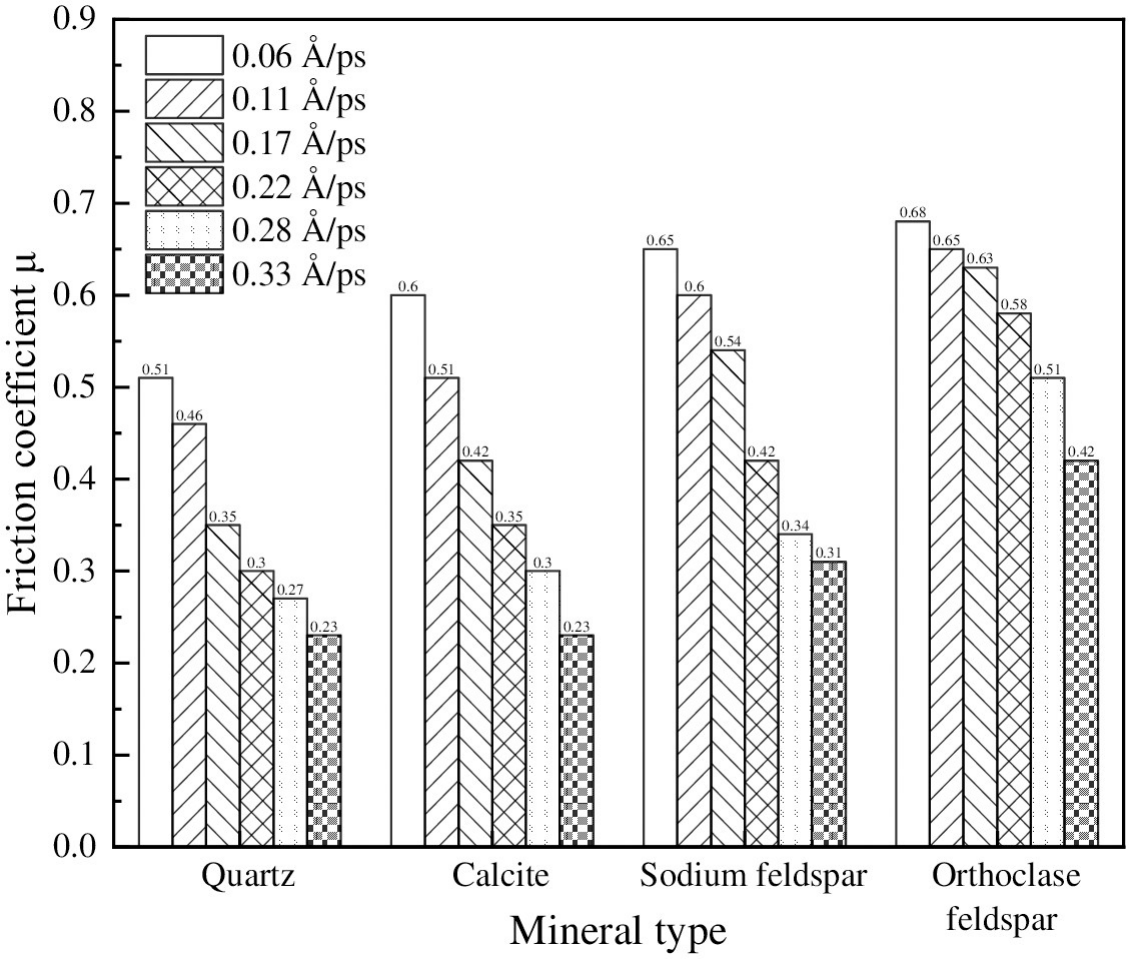
Friction coefficients between minerals and rubber at different shear rates.

To study the influence of different shear rates on the friction coefficients between mineral and NR molecular models, different shear rates need to be determined. Typically, vehicle speeds can be divided into 20 km/h, 40 km/h, 60 km/h, 80 km/h, 100 km/h, and 120 km/h, which correspond to molecular-level velocities of 0.06Å/ps, 0.11Å/ps, 0.17Å/ps, 0.22 Å/ps, 0.28Å/ps, and 0.33Å/ps [34]. The variation of friction coefficients between the mineral-NR-mineral three-layer model at different shear rates is shown in Fig 19.

The presented graph highlighted the notable impact of shear rate on the friction coefficients between the mineral cell and natural rubber cell. It could be observed that the trend of friction coefficient variation with speed remained consistent across different mineral types and natural rubber. Specifically, higher shear rates corresponded to lower friction coefficients between minerals and natural rubber. This indicated that at lower vehicle speeds, there were heightened intermolecular interactions between the tire and mineral molecules, resulting in elevated friction coefficients. However, as vehicle speeds increased, the influence of speed as the primary factor affecting the friction coefficient diminished. Consequently, the intermolecular interactions between the tire and minerals decreased, leading to a reduction in their influence on the friction coefficient.

### 5.4. Correlation analysis

Based on the above research, it is known that the main minerals constituting different types of aggregates are quartz, calcite, and feldspar, and the friction coefficients between different minerals and rubber are different. Therefore, assuming that the adhesive friction coefficient between coarse aggregates and rubber can be linearly superimposed by the adhesive friction coefficients between the main minerals constituting the aggregates and rubber, the adhesive friction coefficient between aggregates and rubber can be predicted based on the adhesive friction coefficients and composition ratios of the main minerals and rubber. According to this assumption, the predicted formula for calculating the AFCAT is shown in Eq (6), and the calculation results are shown in Table 3.


$$AFCAT=\sum \left(\mu_{j}\times P_{j}\right)$$ (6)

**Table 3:** Microscopic adhesive friction coefficients between aggregate and tire.

Aggregate type	Parameter setting value
Basalt	0.61
Limestone	0.52
Granite	0.42

In the above equation, represents the adhesive friction coefficient between a specific primary mineral and rubber; corresponds to the mass percentage (%) of a specific mineral as the primary mineral.

As presented in Table 3, it could be observed that there were notable disparities in the micro-scale adhesion friction coefficients among various types of coarse aggregates and rubber. Specifically, the micro-scale adhesion friction coefficient followed the order: basalt > limestone > granite. This trend in AFCAT aligned with the corresponding trend in macro-scale adhesion friction coefficient (*μ*), as evident from Fig 6. In simpler terms, the adhesion friction coefficient was the highest between basalt aggregate and rubber, while it was the lowest between granite aggregate and rubber. Furthermore, correlation analysis between the obtained AFCAT values and the friction coefficient values (μ) of the stone plate yields a correlation coefficient of 0.81, which is indicating a significant correlation.

## 6. Discussion and conclusions

The findings revealed that the ATCR value of the SMA-13 grade exceeded that of the AC-13 grade, measuring 41.18% and 38.44% respectively. These results emphasize the importance of investigating the adhesive friction performance between the aggregate and rubber.

The macroscopic adhesive friction coefficients μ between rubber and slabs of limestone, basalt, and granite were measured using a pendulum friction tester. The findings indicated significant variations in these coefficients among different aggregate types and rubber. Specifically, the coefficients followed the order: basalt > limestone > granite.

This study examined the micro-scale adhesive friction coefficients between rubber and four major minerals. The results consistently demonstrated a hierarchical order of these coefficients among the minerals at different velocities: feldspar > calcite > albite > aragonite > quartz. Significantly, increasing the shear velocity effectively reduced the adhesive friction coefficient between minerals and rubber. Moreover, the use of constrained shear work facilitated the understanding of the impact of adhesive friction in various mineral-rubber combinations. Given the mineral composition and frictional performance, it is recommended to utilize aggregates with a higher feldspar content and avoid those with a high quartz content.

The AFCAT values, calculated based on the primary minerals present, provided insight into the interaction between coarse aggregates and rubber. The order of AFCAT values was observed as follows: basalt > limestone > granite. Importantly, there was a strong correlation (*r =* 0.81) between AFCAT and μ, affirming the validity of this parameter. Taking into account the adhesive friction performance at both macro and micro scales, it is advisable to utilize basalt aggregates and avoid granite aggregates in the functional layer of the pavement.

This study employed molecular dynamics to examine the frictional interaction between four major minerals and natural rubber. However, it is important to acknowledge that aggregates and rubber used in tire manufacturing encompass a wide range of mineral compositions. Therefore, further research encompassing various minerals and types of rubber is essential.

## References

[1] Pomoni M., et al., Investigation of pavement skid resistance and macrotexture on a long-term basis. International Journal of Pavement Engineering, 2022, 23(4): 1060–1069.

[2] Wu X., et al., The analysis of the factors affecting the macrotexture of bauxite clinker aggregate gradation. Construction and Building Materials, 2020, 244: 118334.

[3] Zhao W., et al., Skid resistance of cement concrete pavement in highway tunnel: A review. Construction and Building Materials, 2023, 406, 133235.

[4] Wang H., et al., Quantifying greenhouse gas emission of asphalt pavement preservation at construction and use stages using life-cycle assessment. International Journal of Sustainable Transportation, 2020, 14(1): 25–34.

[5] James E. and Russo B.J., Analysis of factors affecting injury severity in traffic crashes on Arizona tribal lands. Transportation research record, 2019, 2673(9), 345–354.

[6] Khan I.U. and Vachal K., Factors affecting injury severity of single-vehicle rollover crashes in the United States. Traffic injury prevention, 2020, 21(1): 66–71. doi: 10.1080/15389588.2019.1696962 31906717

[7] Yu M., et al., Dynamic friction coefficient between tire and compacted asphalt mixtures using tire-pavement dynamic friction analyzer. Construction and Building Materials, 2020, 258: 119492.7.

[8] Jamshidi A., et al., Functional and field performance of epoxy asphalt technology–state-of-the-art. Road Materials and Pavement Design, 2023, 24(4): 881–918.

[9] Xiong R., et al., Investigation on anti-skid performance of asphalt mixture composed of calcined bauxite and limestone aggregate. Construction and Building Materials, 2021, 306, 124932.

[10] Wang Z., et al., Formulation of a new warm-mix recycling agent and its rejuvenating effect on aged asphalt. Construction and Building Materials, 2020, 262, 120804.

[11] Jalalkamali R., et al., An investigation of the relationship among skid resistance, mean texture depth and abrasion resistance for different macrotextures of concrete pavements. Civil Engineering Infrastructures Journal, 2021, 54(2): 301–317.

[12] Wu S., et al., Effect of morphological characteristics of aggregate on the performance of pervious concrete. Construction and Building Materials, 2023,367: 130219.

[13] Dong Y., et al., Influence of morphological characteristics of coarse aggregates on skid resistance of asphalt pavement. Materials, 2023, 16(14): 4926. doi: 10.3390/ma16144926 37512200 PMC10381446

[14] Wang H., et al., Correlate aggregate angularity characteristics to the skid resistance of asphalt pavement based on image analysis technology. Construction and Building Materials, 2020, 242: 118150.

[15] Ergin B, et al., Effect of aggregate microtexture losses on skid resistance: Laboratory-based assessment on chip seals. Journal of Materials in Civil Engineering, 2020, 32(4): 04020040.

[16] Zaumanis M., et al., Performance-based design of asphalt mixtures and review of key parameters[J]. Materials & Design, 2018, 141: 185–201.

[17] Pomoni M., et al., Investigation of pavement skid resistance and macrotexture on a long-term basis. International Journal of Pavement Engineering, 2022, 23(4): 1060–1069.

[18] Yao H., et al., Discussion on molecular dynamics (MD) simulations of the asphalt materials. Advances in Colloid and Interface Science, 2022, 299: 102565. doi: 10.1016/j.cis.2021.102565 34871943

[19] Long Z., et al., Analysis of interfacial adhesion properties of nano-silica modified asphalt mixtures using molecular dynamics simulation. Construction and Building Materials, 2020, 255: 119354.

[20] Huang M., et al., Study of diffusion characteristics of asphalt–aggregate interface with molecular dynamics simulation. International Journal of Pavement Engineering, 2021, 22(3): 319–330.

[21] Xu J., et al., Review of interfacial adhesion between asphalt and aggregate based on molecular dynamics. Construction and Building Materials, 2023, 362: 129642.

[22] Zhao G. K., et al., Binary blends of Eucommia ulmoides gum and Nitrile butadiene rubber based on Materials Studio: Compatibility prediction, preparation and properties characterization. Industrial Crops and Products, 2023, 204, 117255.

[23] JTG3450-2019. Field Test Methods of Highway Subgrade and Pavement. Highway & Transportation Industry Standard: 2019, Beijing, China.

[24] Zhang J.K., Study on the Interaction Behavior and Thixotropy of Composite Modified Asphalt Based on Rheology and Molecular Dynamics Methods [D]. Southwest Jiaotong University, 2021.

[25] Cao H.P., Molecular dynamics simulation of mass transfer mechanism of aggregate chemical composition/emulsifier. Chongqing Jiaotong University, 2017.

[26] Guo F., et al., Study on the mechanical properties of rubber asphalt by molecular dynamics simulation. Journal of molecular modeling, 2019, 25, 1–8. 10.1007/s00894-019-4250-x 31776794

[27] Zhuang C.Q., et al., Molecular simulation of the glass transition temperature for Guatta-Percha. Journal of Functional Polymers, 2010, 23 (4): 411–414.

[28] Feng P., et al., Effects of surface texture and its mineral composition on interfacial behavior between asphalt binder and coarse aggregate. Construction and building materials, 2020, 262, 120869.

[29] Guo F., et al., Investigating the interaction behavior between asphalt binder and rubber in rubber asphalt by molecular dynamics simulation. Construction and Building Materials, 2020, 252, 118956.

[30] Yuan X. Q., et al., Molecular Simulation on Miscibility of NR/BR Blends. Chinese Journal of Tropical Crops, 2022, 43(11): 2215–2223.

[31] Ibrahim H., et al., Devulcanized Tire Rubber–Waste Plastic Compounds: A Solution to Improve Storage Stability of Plastic-Modified Bitumen[J]. International Journal of Pavement Research and Technology, 2023: 1–15.

[32] Guo M. and Zhou X., Tire-Pavement Contact Stress Characteristics and Critical Slip Ratio at Multiple Working Conditions. Advances in Materials Science and Engineering, 2019, 2019(1): 5178516.

[33] Gao Y., et al., Impact of minerals and water on bitumen-mineral adhesion and debonding behaviours using molecular dynamics simulations. Construction and Building Materials, 2018, 171, 214–222.

[34] Sun F., et al., A molecular dynamics (MD) simulation on tire-aggregate friction[J]. International Journal of Pavement Research and Technology, 2017, 10(4): 343–351.

